# Imaging-Based Machine Learning Analysis of Patient-Derived Tumor Organoid Drug Response

**DOI:** 10.3389/fonc.2021.771173

**Published:** 2021-12-21

**Authors:** Erin R. Spiller, Nolan Ung, Seungil Kim, Katherin Patsch, Roy Lau, Carly Strelez, Chirag Doshi, Sarah Choung, Brandon Choi, Edwin Francisco Juarez Rosales, Heinz-Josef Lenz, Naim Matasci, Shannon M. Mumenthaler

**Affiliations:** ^1^ Lawrence J. Ellison Institute for Transformative Medicine of USC, Los Angeles, CA, United States; ^2^ Department of Medicine, University of California San Diego, La Jolla, CA, United States; ^3^ Division of Medical Oncology, Norris Comprehensive Cancer Center, Keck School of Medicine, University of Southern California, Los Angeles, CA, United States

**Keywords:** patient-derived organoids (PDO), high content imaging, label-free analysis, machine learning, drug response

## Abstract

Three-quarters of compounds that enter clinical trials fail to make it to market due to safety or efficacy concerns. This statistic strongly suggests a need for better screening methods that result in improved translatability of compounds during the preclinical testing period. Patient-derived organoids have been touted as a promising 3D preclinical model system to impact the drug discovery pipeline, particularly in oncology. However, assessing drug efficacy in such models poses its own set of challenges, and traditional cell viability readouts fail to leverage some of the advantages that the organoid systems provide. Consequently, phenotypically evaluating complex 3D cell culture models remains difficult due to intra- and inter-patient organoid size differences, cellular heterogeneities, and temporal response dynamics. Here, we present an image-based high-content assay that provides object level information on 3D patient-derived tumor organoids without the need for vital dyes. Leveraging computer vision, we segment and define organoids as independent regions of interest and obtain morphometric and textural information per organoid. By acquiring brightfield images at different timepoints in a robust, non-destructive manner, we can track the dynamic response of individual organoids to various drugs. Furthermore, to simplify the analysis of the resulting large, complex data files, we developed a web-based data visualization tool, the Organoizer, that is available for public use. Our work demonstrates the feasibility and utility of using imaging, computer vision and machine learning to determine the vital status of individual patient-derived organoids without relying upon vital dyes, thus taking advantage of the characteristics offered by this preclinical model system.

## Introduction

High-throughput screening assays have advanced the drug-discovery field by greatly increasing the number of compounds that can be screened and thus the number of positive leads. However, this improvement has yet to produce a corresponding increase in the drugs available for treatment as three-quarters of the drugs that enter clinical trials never make it to market, with a majority failing due to a lack of efficacy (1, 2). Oncology drugs have proven especially challenging, with a predicted success rate of only a 3.4% in clinical trials (3). One important limitation of traditional *in vitro* cancer drug screening methods is the use of oversimplified, immortalized cell lines cultured in 2D, which fails to capture the *in vivo* complexity of human tumors including influences from the surrounding microenvironment and cellular heterogeneity (4–6). To improve the success rate of identifying compounds with promising clinical translation, there is a need for more biomimetic preclinical platforms to carry out these drug testing studies. In this context, patient-derived organoids (PDOs), in which cells obtained from a patient’s tumor are grown in a medium that promotes the formation of cellular aggregates that recapitulate important aspects of the original tissue architecture, have gained significant traction in the cancer research field (7–9). Multiple organoid models of human cancers have been developed (10), including gastrointestinal (11), prostate (12), ovarian (13) and pancreatic cancers (14). By more faithfully representing the original physiological environment, these tumor organoid models address some of the limitations of traditional cell line cultures and offer rapid, scalable approaches for patient-specific molecular and phenotypic characterization as well as drug screening (11, 15–17).

Two traditional screening methods typically used to determine compound efficacy are ATP based cell viability assays (18) and vital dyes (VDs) (19). While valuable, both approaches have significant drawbacks in the context of 3D organoid screening. ATP based cell viability assays are disruptive and performed on a pooled population of organoids: as such they do not allow for repeated assaying and mask intra-organoid heterogeneity. Unlike cell viability assays, vital-dye assays are imaging-based and non-disruptive, and therefore, in principle, allow for analysis at multiple timepoints and preserve heterogeneity. However, vital dyes present two significant issues, depending on the specific dye. First, they can have cytotoxic effects and interfere with the outcome readout and, second, they can have transient expression, meaning that the signal indicating a dead cell might peak at a certain timepoint and disappear afterwards. In addition, the per-cell vital dye signal needs to be integrated across multiple cells to obtain a per-organoid viability determination.

Therefore, we propose a label-free high content screening (HCS) method that involves live-cell imaging of colorectal cancer (CRC) patient-derived organoids over time in a robust, non-destructive manner. This approach provides an automated pipeline to visualize cellular dynamics and extract multi-parametric data, which is advantageous for phenotypic screening of PDO models (20). One challenge of HCS platforms is the vast amount of data produced that must be accurately interpreted. To circumvent this bottleneck in analysis pipelines, machine learning (ML) methods can be applied to these large-scale biological data sets. The usefulness of Supervised ML approaches such as linear classifiers and regression models has been demonstrated in analyzing large amounts of data in disparate fields and they are now being used increasingly in the biomedical domain (21–24). Furthermore, computer vision applications can be applied to HCS image data to recognize patterns and changes that are not detectable by the human eye and thus have a huge potential to streamline drug discovery pipelines through screening at a faster pace (25).

Previously, we have shown that imaging-based HCS assays can provide dynamic insight to changes in heterogeneous cellular populations using 2D culture models with a cell-based image analysis method (26). In this study, we trained a linear classifier to discriminate between live and dead PDOs based on a set of morphological and textural features extracted from brightfield images, and then used the trained model to determine drug response of organoids derived from colon cancer patients with heterogeneous clinical histories. Additionally, by collecting the vital status of individual organoids over time we can gain insights into the dynamic aspects of drug response as well as the heterogeneity of response across organoids and across patients. This work showcases the possibilities offered by the application of machine learning approaches to label-free high-content imaging assays.

## Results

### Generation of a Label-Free Imaging-Based Workflow to Evaluate Patient-Derived Organoids

**Figure 1:**
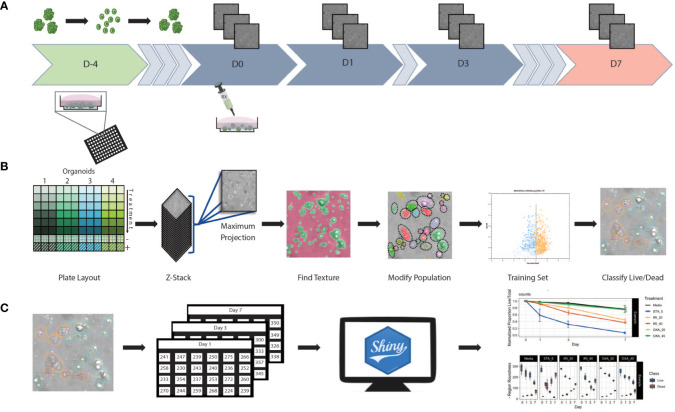
Workflow schematic **(A)** At day -4 organoids were digested to single cells and seeded at 5,000 cells/well in Basement Membrane Extract. Plates were incubated for 4 days allowing organoids to reform. Baseline images were taken at day 0 prior to the initiation of treatment. After initial treatment, plates were re-imaged at days 1, 3, and 7. Media and treatment are refreshed post imaging on day 3, with final measurements taken at day 7. **(B)** Using 96 well plates, multiple patients and/or treatments can be performed with a single assay. Images were obtained in z-stack then combined into a single maximum projection image upon which all further processing and analysis was performed. A textural algorithm was used to identify organoid regions of interest, with a segmenting algorithm applied to split organoids in near proximity to each other. A training set was created by identifying live/dead organoids across untreated and treated samples from each patient. This supervised machine learning algorithm was then applied to experimental data. **(C)** Classification data was compiled into spreadsheets then uploaded to a web-based app for data processing and visualization.

We established an HCS pipeline that includes label-free temporal imaging of PDOs (**Figure 1A**) coupled with quantitative image analysis using a linear classifier (**Figure 1B**) and data compilation and visualization (**Figure 1C**). To execute this workflow, we utilized PDOs from our biobank generated from CRC patient samples, which includes primary and liver metastatic tumors.

*Organoid set up* (**Figure 1A**): Briefly, to generate PDOs that recapitulate the morphology of the tissue of origin (**Supplemental Figure 1**), tissue samples were obtained post-surgery, processed, seeded in extracellular matrix, and expanded for future use (see *Methods* section for details). To set up the screening assay, organoids were first digested to a single cell suspension before being seeded into a 96 well plate. Then, using an HCS platform, the samples were imaged at multiple timepoints in brightfield to minimize phototoxicity and photobleaching.

**Figure 2:**
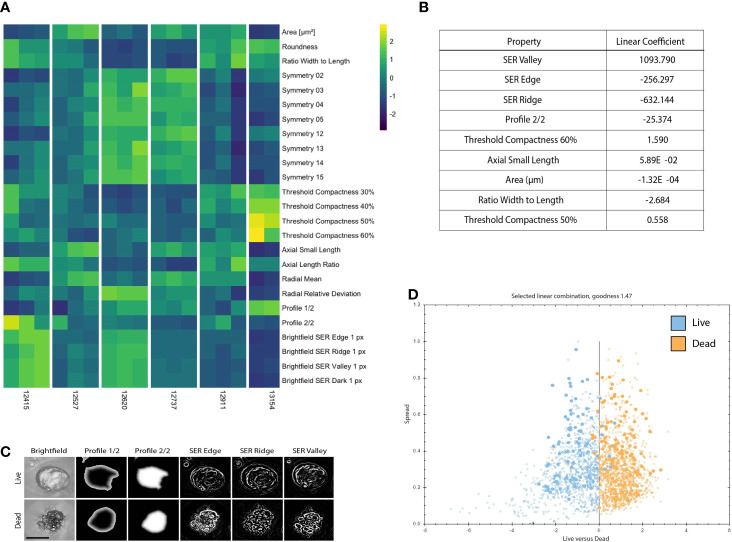
PDOs display distinct texture and morphology features related to viability. **(A)** Heatmap illustrating 25 morphology and texture features (z-score normalized) across 6 different PDOs in the untreated (media only) condition on Day 3. Columns represent replicates for each PDO. **(B)** Features discriminating between live and dead PDOs are listed in order of relevance as indicated by the linear coefficient. **(C)** Representative PDO images of selected morphology and texture features that discriminate between live and dead classification. Scale bar is 50 μm. **(D)** The signal to noise ratio is displayed as goodness of live (blue) versus dead (orange) PDOs manually classified in the training set. Filled circles denote PDOs included in the training set and open circles are classified by the algorithm.

*Supervised machine learning algorithm used to classify organoids as live/dead based on phenotypic features* (**Figure 1B**): Image analysis was subsequently performed on the maximum intensity projections of multiple z-scan images using a machine learning algorithm that enables users to build a linear classifier by identifying regions of interest (ROIs) that are part of distinct groups. Using a trained feature-based textural machine learning algorithm we divided image regions into two classes: organoid ROIs and background (the ML algorithm was trained on the segmented and annotated images, see the *Methods* section for details). Morphological and textural features were measured for each identified object within the organoid class (**Supplemental Table 1**). STAR morphology features encompass Symmetry properties, Threshold compactness, Axial and Radial properties. Spot-Edge-Ridge (SER) textural features are based on Gaussian derivative images measuring pixel intensity patterns within each ROI. Distributions of all 25 STAR and SER features measured across 6 different PDOs, in media or treated with staurosporine (positive control - apoptosis inducer), are depicted in **Supplemental Figure 2** (see PDO counts and tissue site in **Supplemental Table 2**). At day 3, morphological features are patient-specific and consistent across replicate wells (**Figure 2A**) and unsupervised clustering of the data identified clusters that matched the patient or origin rather than number of days in culture (**Supplemental Figure 3**). Using this ML approach, we can detect morphometric similarities and differences across PDOs.

*Statistical analysis and development of data visualization tool* (**Figure 1C**): For all 25 textural and morphological features, the measurements for each detected PDO were first summarized (mean value) across all detected objects within a well. Next, the mean and standard deviation were computed from technical replicate wells for each treatment and time-point. A Shiny-based web tool, the Organoizer, was developed to process the data and produce plots for organoid-survival and monitor changes in features over the course of the treatment period (27).

### Phenotypic Signatures Correlate to PDO Viability

To create a training data set, we manually classified 179 objects, 80 live (untreated media control) and 99 dead (5 μM staurosporine treated positive control), across 41 images of organoids derived from six different patients at various time points. When applied to new experimental data each detected PDO was assigned to either the “dead” or “live” class by the linear classifier based on 9 significant morphology and texture features chosen by the algorithm to delineate between live and dead PDO categories: SER Valley, SER Edge, SER Ridge, Profile 2/2, Threshold Compactness 60%, Axial Small Length, Area, Ration Width to Length, and Threshold Compactness 50% (**Figure 2B**). Representative images illustrate selected PDO textural and morphological features (i.e., Profile 1/2, and 2/2, SER Edge Ridge and Valley), found to be distinct between live and dead PDOs (**Figure 2C**). The signal to noise ratio of the classifier algorithm is expressed as “goodness” based on the distance of the data points from the classifier line, which is visualized using a scatter plot (**Figure 2D**).

**Figure 3:**
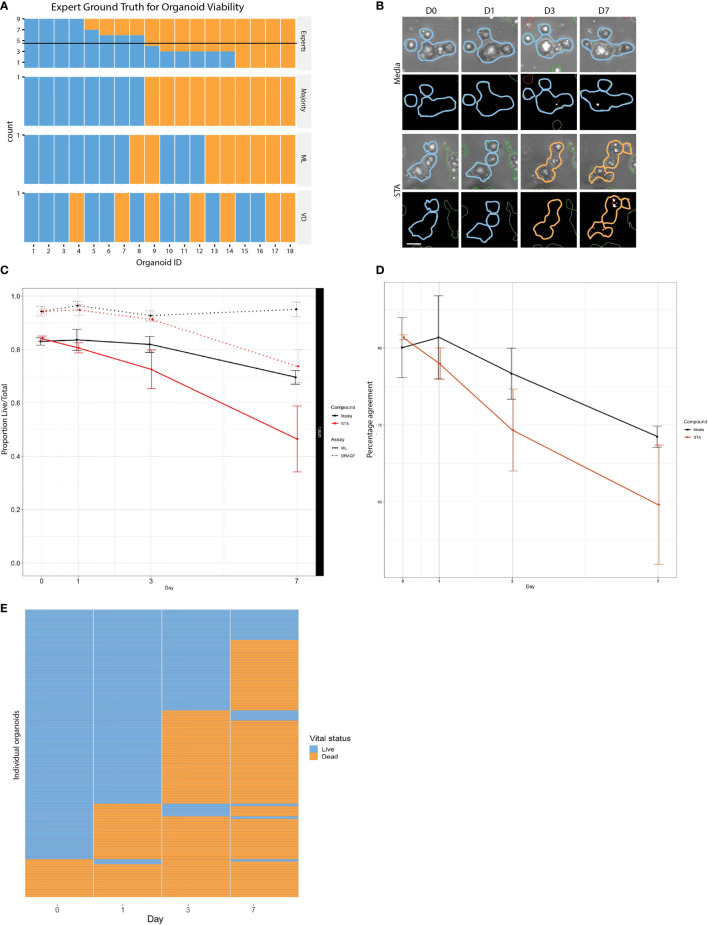
Comparison of live and dead classification by ML and vital dye. **(A)** Classification of 18 different organoid images as either live or dead was determined using three independent methods: tissue culture experts, ML, and DRAQ7, and then compared to determine level of concordance. **(B)** PDOs treated with staurosporine or untreated controls stained with DRAQ7 and classified by ML. Scale bar is 50µm. **(C)** The normalized proportion of live/total organoids (PDO 12620) for the control and staurosporine treated group as determined by both ML and DRAQ7 was plotted over the course of 7 days (error bars: SD of 3 replicate wells per group per timepoint). When classified by ML the difference in response between the treated and untreated groups are seen starting on day 1, whereas VD classification does not start to show separation until after day 3. **(D)** Percentage agreement of ML and DRAQ7 live/dead classification for untreated and staurosporine treated organoids (PDO 12620; error bars are SD of 3 replicate wells each). **(E)** Tracking the vital status of individual organoids (PDO 13154) over 7 days treatment with staurosporine as assessed by our ML classification (N=114 organoids).

As a baseline, we compared our classifier to the visual assessments of trained cell biology experts. Trained cell biologists are adept at visually assessing the health of their cell cultures using brightfield microscopy, however manual classification not only limits throughput but also introduces inter-observer variability (23). To generate a ground truth by visual assessment, we asked 9 scientists to blindly classify images of individual organoids (18 PDOs) as either “live” or “dead” (**Supplemental Figure 4**). Statistical evaluation of inter-rater reliability indicated only moderate agreement between visual classifications (Fleiss’ κ = 0.451, z = 11.5, p = 0.00; perfect agreement κ=1), highlighting the inherent variability in subjective manual classification. Using the data set containing the 18 manually classified organoids, we also performed live/dead classifications based on vital dye (VD) intensity from DRAQ7 staining. For our purposes we defined a dead PDO as one that contained at least one DRAQ7^+^ area ≥ the area of a nucleus. For each organoid we compared the expert consensus classification against DRAQ7 staining results and our linear classifier (**Figure 3A**). We found 78% (14/18) concordance between the linear classifier and expert majority, 61% (11/18) between the linear classifier and DRAQ7, and 61% (11/18) between the expert majority and DRAQ7. In instances where all experts agreed, concordance between the linear classifier and the expert majority increased to 100% (8/8), however expert majority concordance with DRAQ7 only reached 62% (5/8). The strong agreement between the expert classifications and the linear classifier reinforces machine learning as a valuable approach for 3D organoid phenotyping. An important note, the 18 PDO images classified across methods (i.e., experts/ML/VD) were not included in the training set to ensure that there is no “leakage” of information between the training and the testing set.

To further evaluate the performance of the linear classifier using time series data, we compared our algorithm classifications with those made using DRAQ7 for PDO-12620 (**Figure 3B**). We normalized the proportion of live/total PDOs at each timepoint to the proportion of live PDOs at day 0 for untreated and staurosporine treated conditions determined by ML or DRAQ7 staining. For the staurosporine treated group, the ML classifier detected a reduction in the number of live organoids on day 1, whereas DRAQ7 shows a comparable reduction past day 3 (**Figure 3C**). In the untreated group, many organoids are classified as live by both the ML and the VD, with 80% concordance between the two methods. However, the two methods started to diverge upon treatment, with concordance in staurosporine treated organoids dropping to 60% (**Figure 3D**). Additionally, our ML approach allows us to follow individual organoids over time to determine their vital status (**Figure 3E**). To further clarify the discrepancy between the ML-based and DRAQ7-based classifications we tracked individual PDOs (PDO-13154, N~900) over 7 days (days 0,1,3 and 7) and determined their vital status at each time point by each method (**Supplemental Figure 5**). While the ML classification of dead PDOs increased with time, the population of dead PDOs determined by DRAQ7 decreased. The discordance is most likely due to clearance of cellular debris over time where a PDO previously defined as dead is now DRAQ7 negative. Given staurosporine is an inducer of apoptosis, this result suggests that our ML method may identify dead or dying PDOs more accurately including those that have lost their ability to retain DRAQ7.

### Use of Supervised Machine Learning to Track Patient-Specific Drug Response

**Figure 4:**
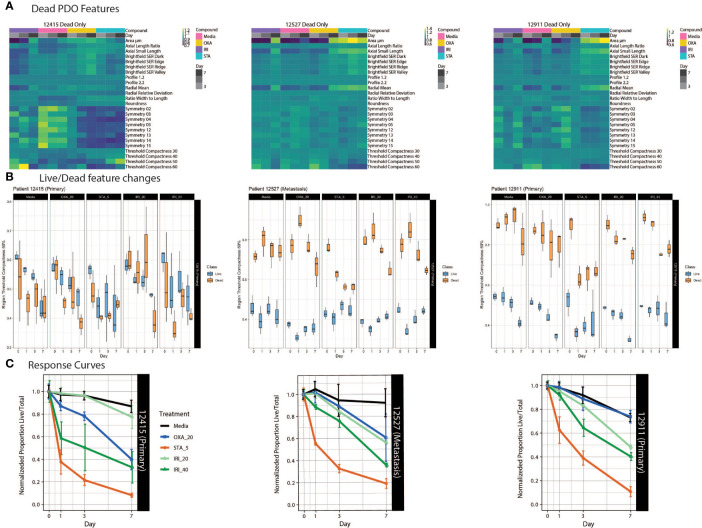
PDO-specific drug responses over time. **(A)** Heat maps of dead PDO features under drug perturbations. Z-score normalized averaged feature values are shown with treatment and time on the x-axis, with features on the y-axis. **(B)** Boxplots of the feature “region threshold compactness 60%” generated using the Organoizer show the variation between the classified live/dead groups. **(C)** Fractions of live/total organoids from untreated media control, irinotecan-treated and oxaliplatin-treated groups are plotted over time to generate dose response curves. Points indicate the mean and bars show the SD.

Tumors evolve over time in response to various stimuli, such as organ-specific microenvironments and drug perturbations. Our approach allows us to characterize the dynamic drug responses of PDOs from both primary and metastatic CRC tumors over time. To accomplish this, we treated PDOs with standard chemotherapy agents: irinotecan, a topoisomerase I inhibitor, and oxaliplatin, an alkylating agent. To interrogate drug specific phenotypic responses, we used heatmaps to examine morphological and textural features within the dead class of PDOs over the course of drug treatment. (**Figure 4A**). Across all PDOs, the feature pattern in the staurosporine-treated group is distinct from the chemotherapy groups. For PDO-12415, the symmetry features in the media control stand out with generally higher feature values compared to the drug treated groups. PDO-12527 and PDO-12911 showed an increase in area and radial mean over time for all treatments, however the symmetry properties of all the PDOs did not show distinct variation across treatment or time. Importantly, the increase in area and radial mean could be attributed to the loss of structure and spreading of dead organoids in response to drug rather than proliferation.

Using our Shiny-based visualization tool we generated box plots of extracted features of interest over time (**Figure 4B** and **Supplemental Figure 6**). We chose to highlight Regional Threshold Compactness 60% due to the unique patterns observed across patients; however, our visualization tool is capable of displaying all features captured. In addition, we plotted dose response curves for each drug treatment (**Figure 4C**). While PDO-12415 (bottom left panel) showed a limited response to irinotecan at 20 μM, a much stronger effect was measured with 40 μM irinotecan starting on day 1. Oxaliplatin at 20 μM also elicited a response with the normalized proportion of live/total dropping 60% by day 7. Analysis of PDO-12527 (bottom middle) revealed a similar response to both oxaliplatin and irinotecan at 20 μM, while irinotecan at 40 μM more effectively killed the PDOs. It appeared that PDO-12911 (bottom right) did not respond to oxaliplatin at 20 μM, as the proportion of live/total PDOs was comparable to the negative control across all 7 days. A slight difference in response timing was seen between the two doses of irinotecan, where the 40 μM dose showed a stronger response at day 3 compared to 20 μM, however by day 7 both showed a response below 40%. Despite temporal variations in response, all three PDOs showed a 60-70% reduction in the proportion of live/total PDOs by day 7. Taken together our ML approach identified PDOs that responded to chemotherapy early in the dosing regimen, highlighting the ability to capture patient-specific drug responses.

## Discussion

Given the breadth of biological models used in cancer research, investigations into drug response should span spatial and temporal scales. However, we continue to apply assays such as those measuring ATP-viability that capture a single readout from a sample/well at a fixed timepoint, which results in a limited understanding of the underlying biology. As seen in **Figure 3**, manual classification limits throughput and introduces person-to-person subjectivity. On the other side, VDs proved problematic for determining the viability of 3D organoids - especially once healthy proliferating organoids develop a necrotic core that contains a substantial fraction of dead cells, masking drug effects. This issue is more commonly seen in PDOs that form large structures, which can be the result of rapidly proliferating organoids. Furthermore, dying cells that initially stain positive using VDs, eventually lose their ability to retain the dye and therefore may erroneously be counted as live.

Here we present an object-based image analysis (OBIA) workflow that is designed to fill the gap between cell and population-level analyses, to dynamically interrogate heterogeneous object-based PDOs in response to perturbations including drug treatment. The non-destructive nature of our platform supports temporal monitoring of phenotypic changes, which allows us to capture the appropriate timing of effects. With an OBIA ML approach we can account for variations in inter-PDO samples (size, shape, etc.). With a larger dataset, one could begin to explore possible correlations between organoid features and patient prognosis (28, 29), shedding light on the clinical relevance of these features.

The imaging workflow described herein provides significant advantages; however, it is important to consider the limitations. Imaging consistency plays a large role in the success of a given assay, and deviations in the XY sample placement can influence results when drawing conclusions across timepoints. Patient to patient variability in PDO size influences the parameters needed for proper segmentation and identification of ROIs; therefore, careful consideration needs to be placed on splitting and merging factors during the segmentation algorithm adjustments. Moreover, additional features and/or separate classification algorithms may be needed to accurately separate live and dead PDO categories when treated with diverse classes of drug compounds.

As many biologists are not computer vision or ML experts, analysis platforms that are accessible to non-experts are needed (30, 31). A paper by Falk et al. describes an ImageJ plugin, U-Net, that enables researchers who are not ML experts to benefit from its application to biological data (32). Furthermore, while the computationally intensive parts of the image analysis workflow are done in a reproducible and automated fashion, biologists are still faced with the task of summarizing the data for different timepoints and conditions across thousands of ROIs. To facilitate this step, we have designed an interactive, web-based tool where users can upload the output of the ML analysis and obtain survival curves and feature metrics. Additionally, while the textural and morphological features that best differentiate between live and dead organoids are automatically determined by the linear classifier, it is often useful to be able to visualize differences across all collected features over time. These tools are accessible at http://organoizer.eitm.org and available for download at https://github.com/eitm-org/organoizer.

We focused our attention on the use of a supervised ML linear classifier algorithm to distinguish live versus dead organoids for the purposes of understanding drug response; however, there are many other questions that could be asked using this method. This workflow enables unrestrained exploration of multidimensional features of organoid morphology and texture characteristics to discover new biology within and across patient samples. Here we demonstrate the utility of our ML image analysis method using a smaller sample set; however, this method can be scaled to perform large drug screens on PDOs generated from different cancer types, providing researchers a flexible yet robust platform for posing their own biological questions. Additional artificial intelligence and ML techniques are being applied to image analysis workflows, including unsupervised techniques such as neural networks and deep learning, which recognize outcomes that are not detectable by humans (21–24). Label-free organoid imaging and batch analysis methods using trained neural networks have been developed from several groups (30, 31, 33, 34). Although these approaches provide highly efficient and precise detection, classification, and measurement of organoid objects, these often require programming skills to create a specific code to train the network and process images. Deep learning-based analysis will be very powerful with large datasets, but additional data processing will be needed to extract specific information. Our ML-based method, with linear classifier and data visualization tool, showed great performance with a relatively small patient sample size. Moreover, it generated multiparametric data including patient-specific organoid morphologies and drug responses over time to understand patient heterogeneity. The rise in patient-derived biobanks, combined with sophisticated image analysis techniques using ML approaches, presents a valuable platform for drug screening and discovery.

## Online Methods

### Cell Culture and Reagents

Organoid growth medium consists of base medium (ADMEM/F12 with 10% FBS, 1% penicillin/streptomycin, 1% Glutamax, and 1% HEPES) supplemented with 1X N2 (Sigma Aldrich, 17502048), 1X B-27 (Sigma Aldrich,17504044), 1mM N-Acetylcysteine (Sigma Aldrich, A7250) 50 ng/ml EGF (Life Technologies, PGH 0313), 100 ng/ml Noggin (Tonbo, 21-7075-U500), 10 mM nicotinamide (Sigma, N0636), 500 nM A 83-01 (Calbiochem, 616454-2MG), 10 μM SB202190 (Sigma 47067), and 0.01μM PGE2 (Sigma Aldrich, P5640).

Tissue digestion solution consists of 1.5 mg/ml collagenase (Millipore, 234155), 20 μg/ml hyaluronidase, (MP Biomedicals 100740) and 10 μM Ly27632 (Sigma Y0503).

### Generation and Expansion of Human Colorectal Cancer PDOs

Tumor tissue was received from consented patients following Institutional Review Board (IRB) approval at the Norris Comprehensive Cancer Center of USC, Los Angeles CA. Tissue was washed with PBS, minced and digested for 30 minutes at 37°C. Digest suspension was filtered using a 100 μm strainer to remove large residual pieces of tissue, then centrifuged at 189 x g for five minutes. Pellet was washed in DMEM/F12 media (ThermoFisher, 11320033) supplemented with 10% FBS three times and single cells were re-suspended in BME (Culturex® Reduced Growth Factor Basement Membrane Matrix Type 2, Trevigen, 3533-005-02). Cell/BME mixture was plated in 24 well plates with 60 μl per well and incubated upside down at 37°C until solidified (10-20 minutes). Then 500 μl of organoid growth media was layered on top and media was changed as needed. To passage organoids, BME was dissociated with 500 μl/well TrypLE (ThermoFisher Scientific, 12605028) for 5 minutes at 37°C. Organoid suspension was pooled and centrifuged at 450 x g, the pellet was re-suspended in BME and re-plated in a 24 well plate. PDOs used for experiments were ≤ 20 passages in culture.

### Drug Treatment Studies

PDOs were harvested from BME using Gentle Cell Dissociation Reagent (Stemcell technologies, 07174), pooling all wells and incubated on ice for 45 minutes then centrifuged for 5 minutes at 189 x g. Supernatant was removed and the pellet was re-suspended in 50% TrypLE with 10 µM Y-27632 (Stemcell Technologies, 72302), incubated at 37°C for 10-15 min with occasional agitation. Alternatively, PDOs were harvested using 500 μl/well TrypLE, incubated at 37°C for 30 minutes. For both methods, PDOs were centrifuged for 5 minutes at 189 x g. Supernatant was removed and the pellet was re-suspended in 1 ml of organoid base medium then filtered through a 40 μm strainer to remove aggregates. Flow through was centrifuged at 189 x g for 5 minutes and the pellet was re-suspended in BME. A 96 well μ-Plate (Ibidi, 89646) was coated with 5 μl of BME/well and incubated at 37°C until BME solidified. The μ-plate was then seeded with 5 μl BME/cell mixture at a concentration of 1000 cells/μl and topped with 70 μl organoid growth medium.

#### I. Image-Based Organoid Drug Response Assay

*Image acquisition*. Plates were incubated for four days prior to imaging on the Operetta HCS platform (PerkinElmer). Baseline images were taken on day 0 followed by respective drug treatments: irinotecan (Sigma-Aldrich, I1406), oxaliplatin (Sigma-Aldrich, O9512), staurosporine (Sigma-Aldrich, 569396). Additional images were acquired on days 1, 3, and 7 post drug treatments. Images were acquired in brightfield, with 23 z-stacks ranging from 20-460 μm at increments of 20 μm. On day 3 of the experiment, imaging medium was changed and replaced with fresh medium and drugs.

*Image Analysis*. Z-stack images were combined into single maximum projection images which were then analyzed using Harmony (PerkinElmer) image analysis software. ROIs were generated using the “Find Texture” supervised ML feature. Training areas of 15 pixels, with texture scaling (2 pixels) were used to define the distance, and region scaling (6 pixels) defines the smoothness of region borders. These ROIs were modified as needed per visual analysis using the “Modify Population” feature to achieve optimal splitting of objects. Specifically, further segmentation was performed to partition the organoid area into multiple, distinct class regions corresponding to individual organoids, by applying a hole-filling algorithm followed by a cluster-by-distance method to detect individual objects within clusters. After objects at the border of the image were removed from the analysis set, morphological and textural features of complete organoids (ROIs) were measured and extracted. A final filtering step based on the object area measurement was applied to exclude small debris as well as large, unsegmented organoid clusters from the data set. In addition, the commercially available PhenoLogic™ ML algorithm (PerkinElmer) was used to classify organoids as live or dead.

#### II. VD Dead Cell Labeling, Imaging, and Analysis

DRAQ7 (Biolegend, 424001), at a final concentration of 5 μM, was added to plates 30 minutes prior to imaging on day 0. Additional 5 μM DRAQ7 was added on day 3 along with fresh medium and drug. Images were acquired with excitation at 633 nm. Areas positive for DRAQ7 were detected within each organoid ROI. ROIs containing one or more areas of DRAQ7 were classified as dead.

### Statistical Analysis

Organoid ROIs were counted, and ROI-level morphological metrics were averaged on a per-well basis at each timepoint and for each class (“dead” vs. “live”). The mean and standard deviation were then computed from replicate wells with the same treatment conditions. Response curves were computed as either the proportion of “live’ ROIs over “dead” ROIs (ratio) or as “live” ROIs over all ROIs (proportion). Optionally, the proportion or ratio of “live” organoids can be normalized to the proportion (or ratio, respectively) on the first day of measurement (usually day 0). Boxplots for each feature across timepoints were also generated. The code is available at https://github.com/eitm-org/organoid_drug_response.

Heatmaps were generated by averaging feature values per well, then taking the average value across wells to get one average value for each unique group. Rows and columns were grouped using hierarchical clustering and rows were scaled using the heatmap package in the R statistical computing language. All analyses were performed using the R statistical language (v. 4.1.0) using the following packages: cowplot (v.1.1.1) (35), eulerr (v. 6.1.1) (36), ggridges (v. 0.5.3) (37), ggthemes (v. 4.2.4), here (v. 1.0.1), irr (v. 0.84.1) (38), knitr (v. 1.33), networkD3 (v. 0.4), pheatmap (v. 1.0.12), plater (v. 1.0.3), readxl (v. 1.3.1), reshape2 (v. 1.4.4), scales (v. 1.1.1), tidyverse (v. 1.3.1) and viridis (v. 0.6.1) (27, 39–49).

## Data Availability Statement

The datasets presented in this study can be found in online repositories. The names of the repository/repositories and accession number(s) can be found below: https://github.com/eitm-org/organoid_drug_response.

## Ethics Statement

The studies involving human participants were reviewed and approved by Institutional Review Board at the University of Southern California Norris Comprehensive Cancer Center. The patients/participants provided their written informed consent to participate in this study.

## Author Contributions

ERS performed and analyzed the experiments and wrote the manuscript. NU and EFJR contributed to data analysis and visualization. SK, KP, and BC provided manuscript feedback and assistance with experimental design. CD contributed to image analysis. RL processed patient samples. CS and SC performed preliminary experiments. HJL offered clinical insights and patient samples. NM was responsible for data analysis, visualization, experimental design, and editing of the manuscript. SMM was responsible for study concept and design, interpretation of data, supervision of the study, and editing of the manuscript. All authors contributed to the article and approved the submitted version.

## Funding

This work was funded by a generous donation made by the Stephenson family, who supported research as part of the Stephenson Family Personalized Medicine Center at the Ellison Institute for Transformative Medicine. It was also funded by a SPRC pilot project awarded through the Ellison Institute for Transformative Medicine.

## Conflict of Interest

The authors declare that the research was conducted in the absence of any commercial or financial relationships that could be construed as a potential conflict of interest.

## Publisher’s Note

All claims expressed in this article are solely those of the authors and do not necessarily represent those of their affiliated organizations, or those of the publisher, the editors and the reviewers. Any product that may be evaluated in this article, or claim that may be made by its manufacturer, is not guaranteed or endorsed by the publisher.
