# Supplementary material for: Imaging-Based Machine Learning Analysis of Patient-Derived Tumor Organoid Drug Response
Source: Front Oncol. 2021 Dec 21;11:771173. doi: 10.3389/fonc.2021.771173 (PMC8724556; doi:10.3389/fonc.2021.771173)
Supplement: Supplementary file 1 [file DataSheet_1.pdf]

Supplemental Table 1. Morphological and Textural Feature Extraction

Standard Morphology Features	STAR Morphology Features		SER Textural Features
Region Area (um^2)	Region Symmetry 02	Region Threshold Compactness 40%	Region Brightfield SER Edge 1 px
Region Roundness	Region Symmetry 03	Region Threshold Compactness 50%	Region Brightfield SER Ridge 1 px
Region Ration Width to Length	Region Symmetry 04	Region Threshold Compactness 60%	Region Brightfield SER Valley 1 px
	Region Symmetry 05	Region Axial Small Length	Region Brightfield SER Dark 1 px
	Region Symmetry 12	Region Axial Length Ration	
	Region Symmetry 13	Region Radial Mean	
	Region Symmetry 14	Region Radial Relative Deviation	
	Region Symmetry 15	Region Profile 1/2	
	Region Threshold Compactness 30%	Region Profile 2/2	

Supplemental Table 2. Tissue of origin and number of PDOs evaluated per patient

PDO	Tissue	Count
12415	Primary Colon Cancer	79
12527	Liver Metastases	270
12620	Liver Metastases	267
12737	Primary Colon Cancer	778
12911	Primary Colon Cancer	381
13154	Liver Metastases	332

Supplemental Figure 1

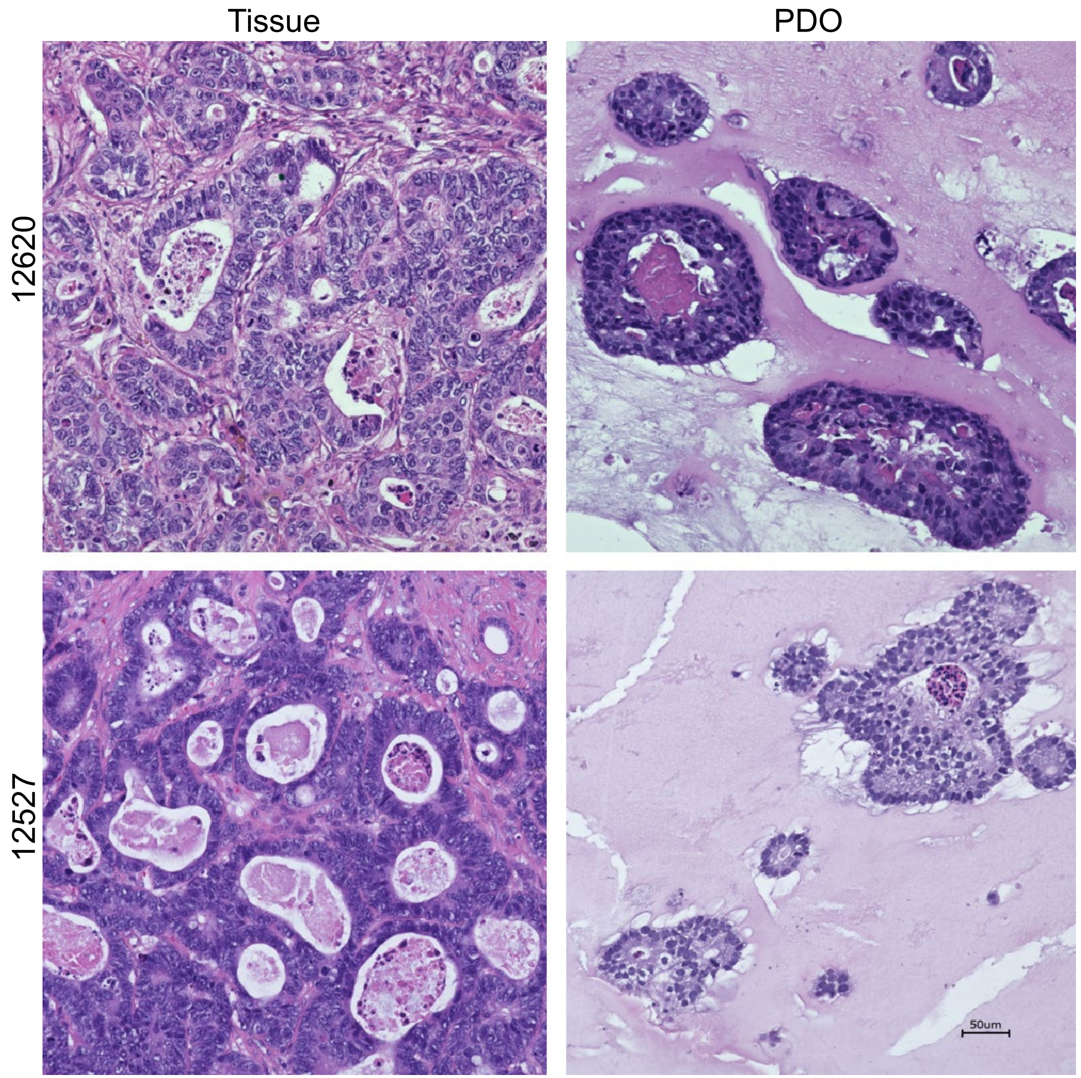

Supplemental Figure 2

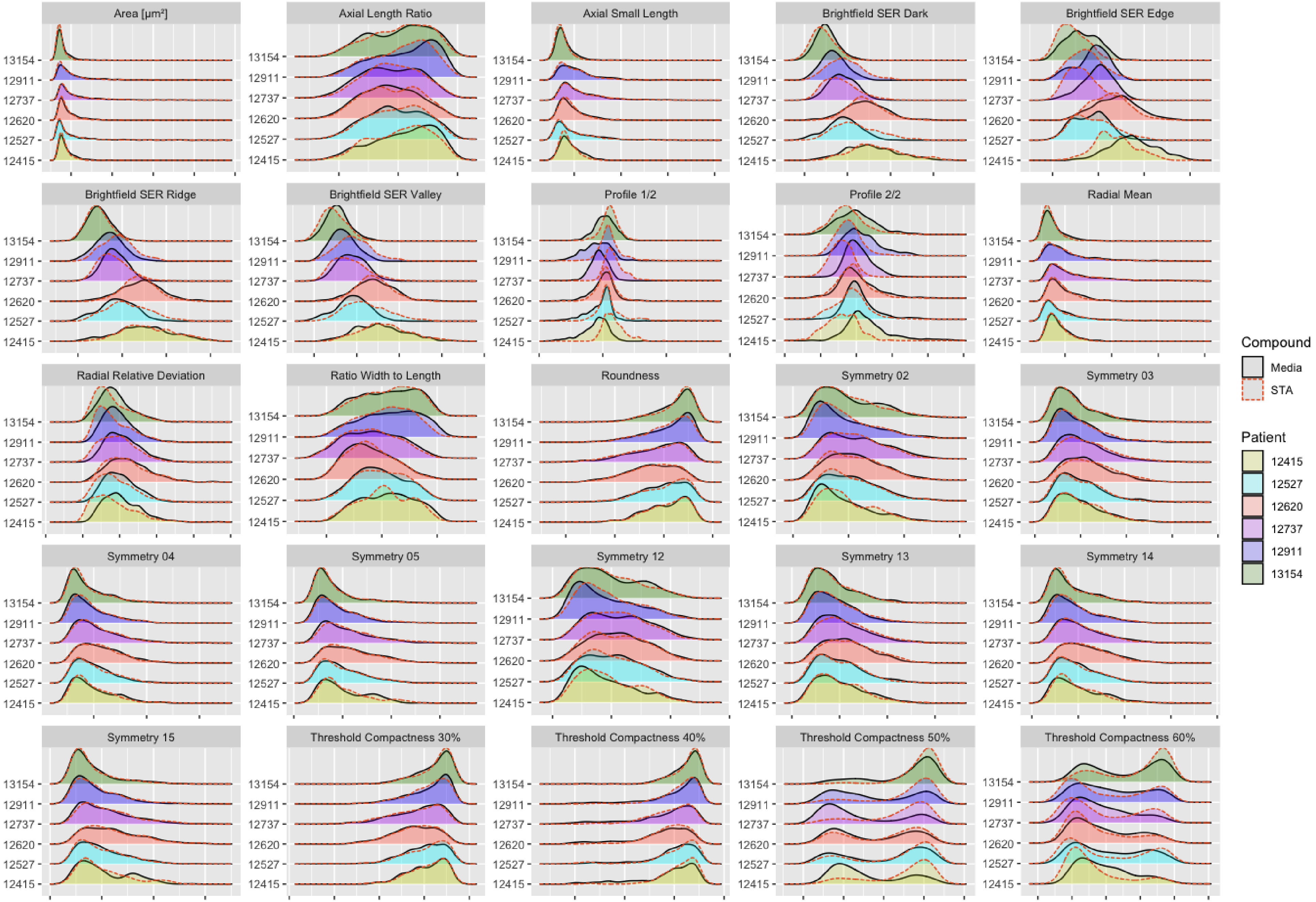

Supplemental Figure 3

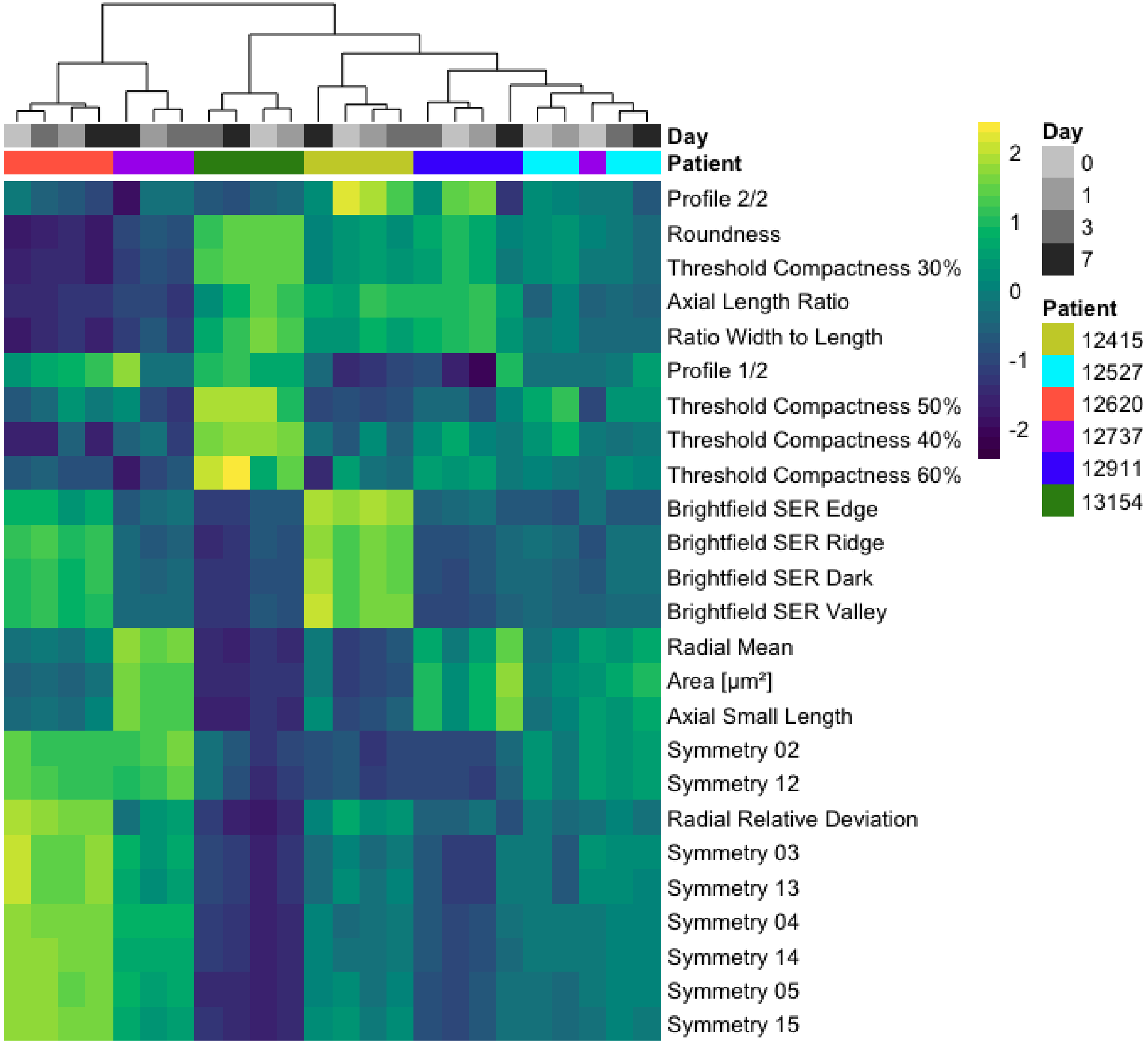

Supplemental Figure 4

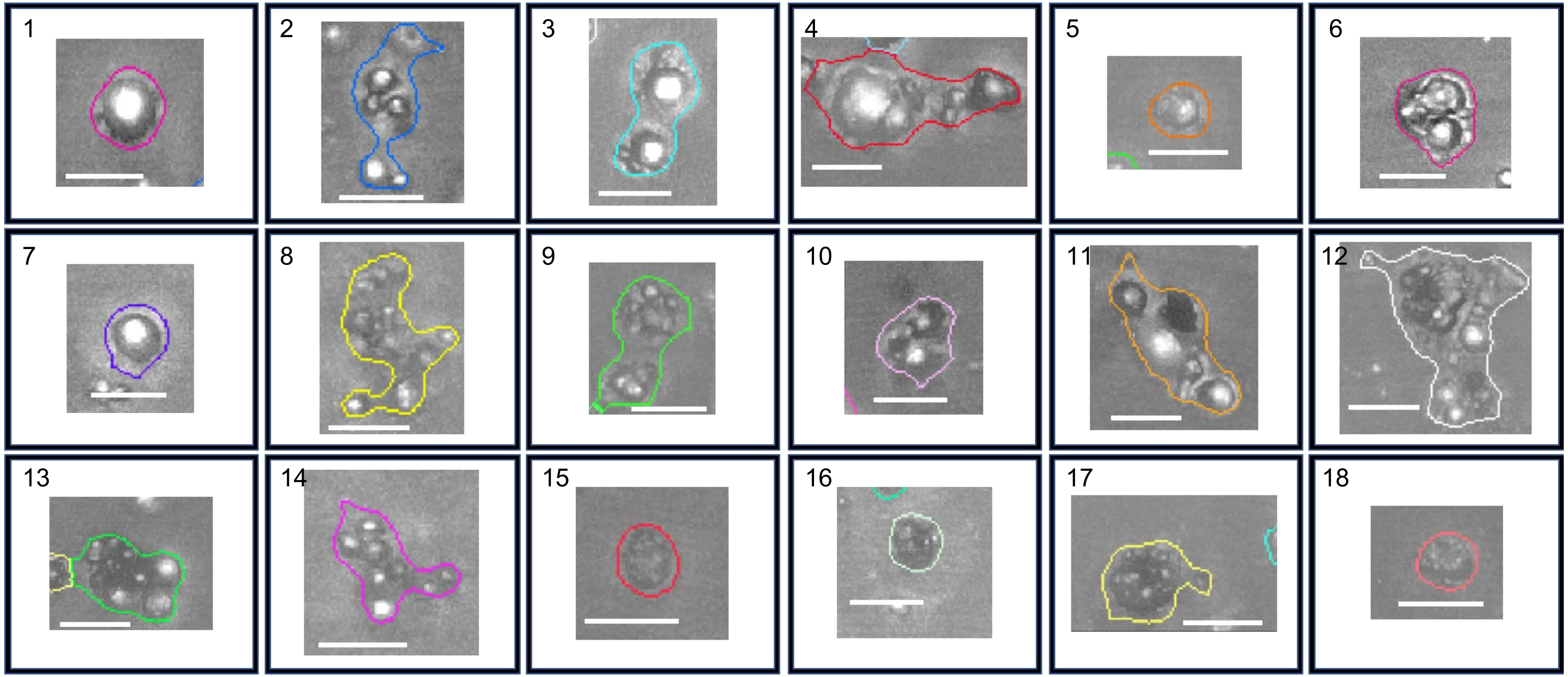

Supplemental Figure 5

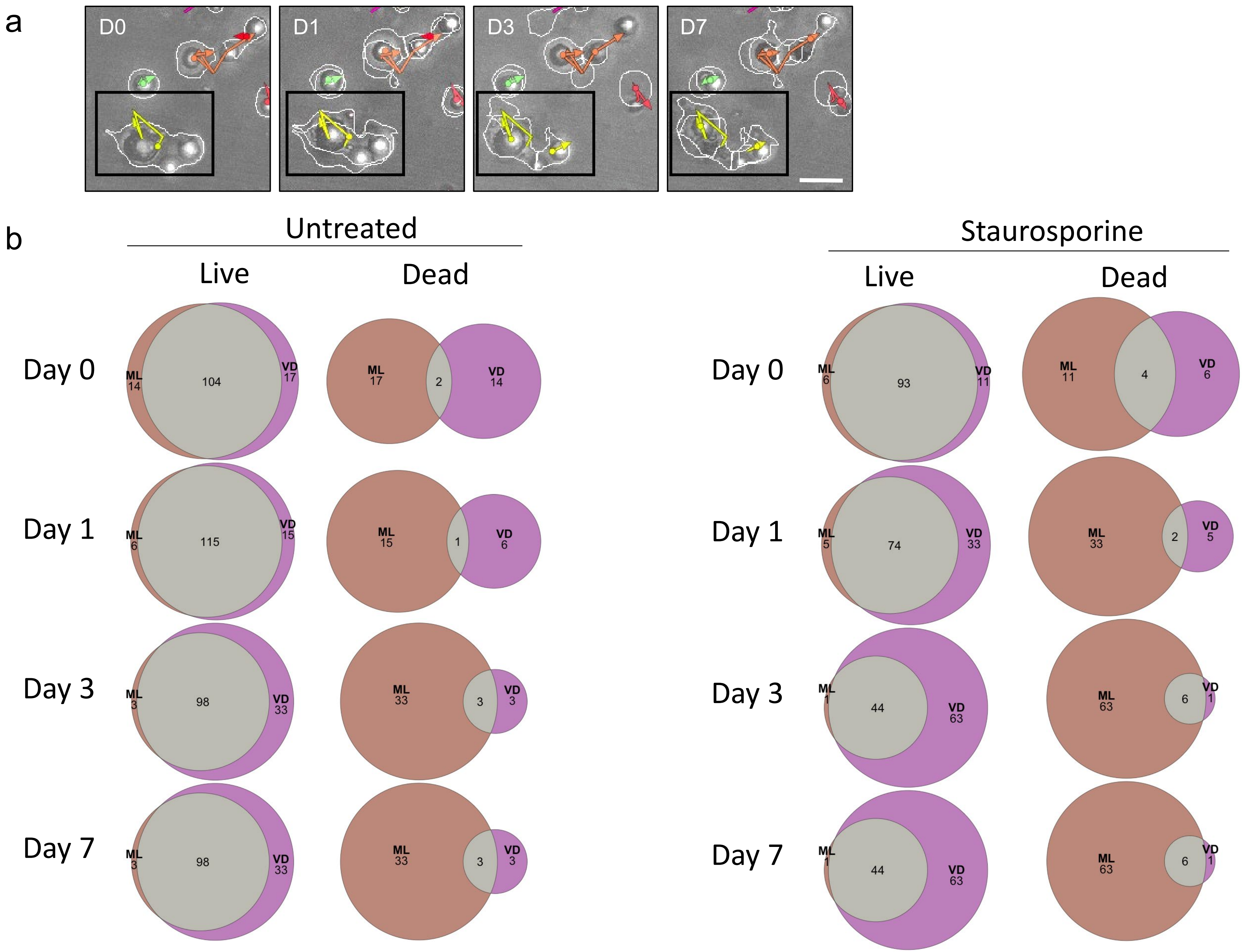

Supplemental Figure 6

a

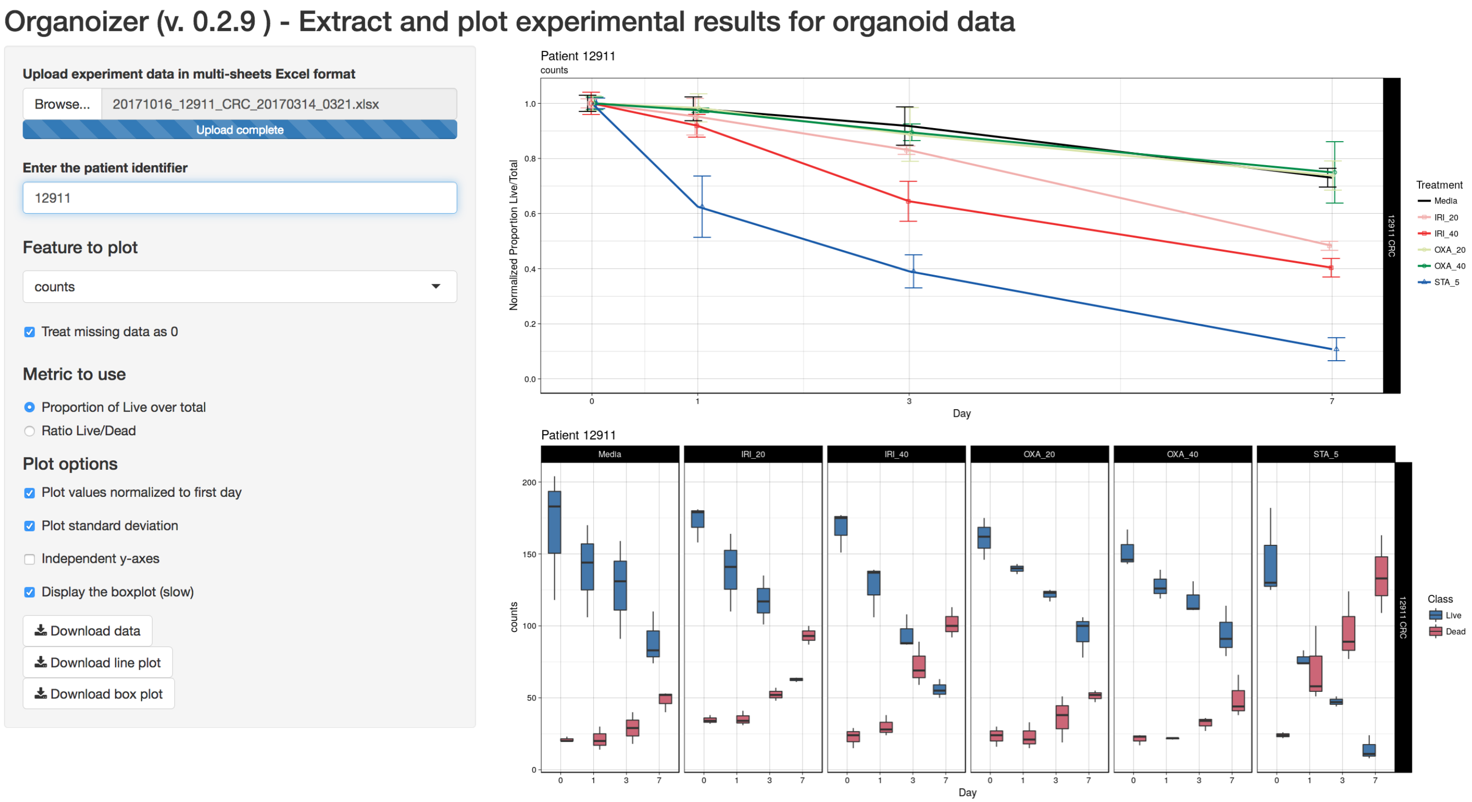

b

	J	K	L	M	N	O	P	Q	R	S	T	U	V	W	X	Y	Z	AA	AB	AC	AD
1	Position X [µm]	Position Y [µm]	Dead - Region Area [µm²]	Dead - Region Roundness	Dead - Region Ratio Width to Length	Dead - Region Symmetry 02	Dead - Region Symmetry 03	Dead - Region Symmetry 04	Dead - Region Symmetry 05	Dead - Region Symmetry 12	Dead - Region Symmetry 13	Dead - Region Symmetry 14	Dead - Region Symmetry 15	Dead - Region Threshold Compactness 30%	Dead - Region Threshold Compactness 40%	Dead - Region Threshold Compactness 50%	Dead - Region Threshold Compactness 60%	Dead - Region Axial Small Length	Dead - Region Axial Length Ratio	Dead - Region Radial Mean	Dead - Region Radial Relative Deviation
2	362	442	2754	0.9234	0.774	0.06325	0.01467	0.05335	0.06112	0.06856	0.03292	0.08101	0.08514	1.041	1.018	0.2532	0.2589	20.49	0.9257	20.09	0.357
3	121	212	3794	0.6966	0.5334	0.05419	0.03897	0.2013	0.08562	0.1146	0.04327	0.3098	0.1272	0.8112	0.4418	0.1703	0.4595	23.89	0.8273	24.43	0.4201
4	127	121	1014	0.8957	0.6794	0.05546	0.1533	0.03839	0.05932	0.08114	0.2239	0.05317	0.08678	1.024	1.024	1.024	1.024	12.46	0.9014	12.32	0.3763
5	499	-422	1657	0.9622	0.8078	0.0829	0.02577	0.03047	0.02147	0.1163	0.0291	0.038	0.02748	1.084	1.084	1.053	0.3157	15.35	0.8595	15.71	0.3522
6	-328	1232	1219	0.9486	0.6667	0.1725	0.02608	0.04552	0.01996	0.2491	0.03623	0.07258	0.0216	1.074	1.074	1.074	1.021	11.83	0.7174	13.43	0.3764
7	-493	1078	2278	0.8773	0.6327	0.1527	0.1545	0.04263	0.05133	0.2319	0.2262	0.07442	0.0911	0.9961	0.9961	0.8716	0.2361	16.72	0.7287	18.62	0.4044
8	-64	896	1025	0.7508	0.363	0.5364	0.09592	0.1869	0.09266	0.654	0.1281	0.2837	0.1335	0.879	0.879	0.879	0.8523	8.742	0.3956	15.4	0.4369
9	379	567	1092	0.975	0.7691	0.09396	0.06415	0.01488	0.02766	0.1382	0.09848	0.0239	0.04384	1.102	1.102	0.9898	0.2848	12.07	0.8309	12.54	0.3675
10	297	561	1551	0.6328	0.4028	0.4348	0.2317	0.04822	0.03458	0.4921	0.2578	0.05918	0.02472	0.7556	0.7556	0.7395	0.3398	14.29	0.5605	19.07	0.4172
11	627	388	2306	0.9661	0.775	0.1029	0.05176	0.02568	0.006152	0.1551	0.07688	0.03575	0.01286	1.085	1.085	1.085	0.9508	17.58	0.8123	18.57	0.3552
12	-340	388	1129	0.9437	0.6329	0.1924	0.05609	0.06936	0.03023	0.2886	0.08848	0.1214	0.05146	1.071	1.071	0.3723	0.3236	11.01	0.6703	13.01	0.394
13	289	360	1013	0.9747	0.8584	0.01592	0.03053	0.008465	0.01771	0.02228	0.04841	0.01145	0.02608	1.103	1.103	1.103	1.061	12.68	0.9713	12.13	0.3538
14	535	347	1312	0.9993	0.8418	0.04701	0.01624	0.02369	0.01302	0.07562	0.0204	0.04037	0.02093	1.124	1.124	1.124	0.7497	13.98	0.9008	13.95	0.3486

D0_Live | D0_Dead | D1_Live | D1_Dead | D3_Live | D3_Dead | D7_Live | D7_Dead | +

Supplemental Figure Legends

Supplemental Figure 1. PDO morphology recapitulates the tissue of origin. Hematoxylin and eosin staining of patient-matched CRC tissues and organoids. Polarized cells and distinctive lumen show morphological similarities between organoids and their respective tissues. Scale bar, 50μM.

Supplemental Figure 2. Distribution of morphology and texture features across PDOs. Ridgeplots illustrate similarities and differences in 25 morphology and texture features across 6 PDOs in untreated and staurosporine-treated conditions.

Supplemental Figure 3. PDOs display patient-specific texture and morphology. Features of PDOs were mapped using unsupervised hierarchical clustering and identified clusters by patient rather than number of days in culture.

Supplemental Figure 4. Expert classification images. Images qualitatively classified as "live" or "dead" by experts. Scale bars at 50 μm.

Supplemental Figure 5. Temporal dynamics of PDOs. (a) Representative multi-timepoint images of PDOs. ROIs are outlined in white, circles indicate the centroid of the ROI, and arrows indicate direction of movement. Scale bars are 50 μm. (b) Untreated and staurosporine-treated PDOs classified as either live or dead by ML and VD from tracked individual PDOs. Venn diagrams show the number of organoids identified as "Live" or "Dead" by each methodology at each day. ML: Machine Learning, VD: Vital Dye.

Supplemental Figure 6. Layout of the Organoizer web tool and data format. (a) Dropdown menu, and display options on the left, with output graphs on the right. Line graphs on right top, and boxplots on bottom right. Example of data formatting for the organoizer (b) Data from each timepoint is separated by live/dead classification and recorded in individual tabs. Within each tab, individual features are separated by columns and each object is separated by rows.
